# Optical Coherence Tomography in Ophthalmology

**DOI:** 10.1155/2012/134569

**Published:** 2012-04-24

**Authors:** Eduardo Buchele Rodrigues, Felipe Medeiros, Stefan Mennel, Fernando M. Penha

**Affiliations:** ^1^Department of Ophthalmology, Federal University of São Paulo, R. Botucatu, 821 Vila Mariana, 04023-062 São Paulo, SP, Brazil; ^2^Department of Ophthalmology, University of California, San Diego, CA 92093, USA; ^3^Department of Ophthalmology, Philipps University Marburg, 35032 Marburg, Germany; ^4^Department of Ophthalmology, Federal University of São Paulo, São Paulo, SP, Brazil

Optical coherence tomography (OCT) is an optical signal acquisition and processing method that captures micrometer-resolution three-dimensional images from within biological tissues. In recent years, OCT has become an important imaging technology used in diagnosing and following macular pathologies. It has complemented fluorescein angiography in many cases, especially in the diagnosis and management of various retinal disorders, including macular edema and age-related macular degeneration. In addition, further development enabled application of optical coherence tomography in evaluation of the integrity of the nerve fiber layer, optic nerve cupping, anterior chamber angle, or corneal topography.

In this special issue, a number of researchers and physicians prepared twelve papers to discuss developments on the application of OCT for ocular diseases diagnosis. It comprises three review articles, six original articles, and three case reports. One of the review articles is an overview on the usefulness of OCT for the anterior segment entities by our work group. The other two review papers deal with the use of OCT in ophthalmic tumors and on the simultaneous confocal scanning laser ophthalmoscopy combined with high-resolution spectral domain. Three case reports show unique features of OCT in patients with fundus abnormalities as diffuse unilateral subacute neuroretinitis, central serous chorioretinopathy, and punctate inner choroidopathy. Original papers on various subjects such as glaucoma diagnostics, retinal detachment, or retinitis pigmentosa offer novel insights into ophthalmology research.

We expect this special issue to be a contribution to the progress in imaging in ophthalmology. The diversity of papers illustrates the wide utility of OCT in ophthalmology. 

